# Supplementary material for: Nurse-Led Interventions in Chronic Obstructive Pulmonary Disease Patients: A Systematic Review and Meta-Analysis
Source: Int J Environ Res Public Health. 2022 Jul 26;19(15):9101. doi: 10.3390/ijerph19159101 (PMC9368558; doi:10.3390/ijerph19159101)
Supplement: Supplementary file 1 [file ijerph-19-09101-s001.zip › ijerph-1812603-supplementary.pdf]

Table S1. Characteristics of studies focusing on nurse-led COPD interventions.

Study	Country	Period	N.	Design	Population	Intervention vs comparison	Outcome variables
**Abad Corpa et al. 2013**	Spain	24 weeks	143<br>GI: 56<br>GC: 87	Quasi-experimental study with a control group, pseudo-random by services	- Patients admitted with a main diagnosis of COPD greater than 18 months.	- Home visits by hospital and primary care nurse coordinated in a team with telephone calls + extra intervention<br>- Hospital evaluation knowledge about COPD and individualized care plan of relative and patient for 5 days<br>vs.<br>- Telephone calls without extra intervention	- Level of satisfaction with nursing care: LOPSS12.<br>- Use of health service after discharge<br>- N. of days admitted after discharge<br>- Assessment health status: Apache II Severity Index<br>- Medication compliance: Morinsky–Green test<br>- Social situation: Gijón scale.<br>- Knowledge therapeutic regimen: Nursing Outcomes Classification (NOC)<br>- Cognitive state: Mini mental.<br>- Severity level: severity index.<br>- Physical disability scale: from Red Cross.<br>- Fragility index of: Katz<br>- Quality of life: SGRQ
**Ahn et al. 2020**	South Korea	6 months 2018-2019	261	Prospective cohort study	- Age >40 years.<br>- Diagnosis of COPD with minimum inhaler use for 1 month	- 3 visits in 6 months after discharge by a nurse specialized in inhalation therapy	- Quality of life: MRC, CAT, EQ 5D, PHQ 9<br>- Satisfaction with inhaler use:<br>- ISF-10

						to train, educate and clarify patient's doubts.<br>vs.<br>- Usual therapy	
**Akinci et al. 2011**	Turkey	3 months 2005-2007	32<br>GI:16<br>GC:16	RCT Randomized controlled trial	- GOLD III, IV.<br>- No history of infections or exacerbation of respiratory symptoms.<br>- No medication changes preceding 2 months.<br>- No myocardial infarctions preceding 4 months.<br>- No serious congestive heart disease<br>- No serious problems of hypertension, diabetes mellitus<br>- With complications and muscle and joint problems.<br>- GOLD III, IV.	- Efficacy of nurse-led home pulmonary rehabilitation<br>vs.<br>- Absence of rehabilitation program.	-Pulmonary function:<br>- Forced expiratory volume in First second (FEV1 % predictive-value)<br>- FEV1 /FVC.<br>- Arterial blood gases:<br>- ABL 700 series.<br>- American Thoracic Society's criteria.<br>- Quality of life (SGRQ).<br>- Dyspnea level: BDI.<br>- Functional capacity:<br>- 6-minute walk test (6MWT)

**Al-Kalaldeh et al. 2016**	Jordan	8 months 2015	121	Quasi-experimental study	- Age> 18 years.<br>- Diagnosed COPD > 2 years with inhaler > 6 months.<br>- Exclusion:<br>- Lung disorders other than COPD.<br>- Dementia, psychosis, or profound deafness	- Health education about inhalers:<br>- Purposes, action and adverse effects.<br>- Assessment of correct use of Inhaler.	- Knowledge inhalers:<br>- Inhaler Proficiency Schedule (IPS).<br>- Behavior before use of Inhaler:<br>- Patient Reported Behavior (PRB)
**Ansari et al. 2020**	Australia	1 1/2 year 2015-2016	44	Experimental study of a single group.	- Patients with COPD.<br>- Age 40 < 84 years.<br>- At least one other chronic condition or coexisting comorbidity.<br>- No cognitive impairment.<br>- Knowledge of English language.	- Patients with COPD activated through personalized self-care support that recognizes implications of comorbidities, generating:<br>- Improved health behavior regarding COPD.<br>- Improve knowledge and self-management of COPD.<br>- Increased self-efficacy in empowerment in general health management.	- Perception of multimorbidity burden: Multimorbidity Illness Perceptions Scale (MULTIPLES).<br>- Patient Activation: Patient Activation Measure (PAM 13).<br>- Impact of COPD on patient's life: - COPD Assessment Test (CAT); COPD Knowledge: - COPD Knowledge Questionnaire (COPDQ).

**Bal Özkaptan et al. 2016**	Turkey	12 months 2012-2013	106 GI:53 GC:53	Randomized clinical trial	- Patients with COPD for at least 1 year.<br>- No difficulty communicating.<br>- No disease except diseases related to COPD.<br>- Resident in city center.	- Home nursing care with self-efficacy self-care model of patients with COPD<br>- Guidelines given on first visit, then 4 visits in 3 months<br>vs.<br>- Home nursing care with self-efficacy self-care model of patients with COPD,<br>- Guidelines given during visit	- Dyspnea:<br>- MRC<br>- COPD self-sufficiency:<br>- COPD Self-efficacy scale<br>- CSES
**Benzo et al. 2019 (7)**	USA	12 months	215 GI:108 GC:107	Randomized controlled trial	- COPD patients<br>- Ability to communicate by phone	- Post-discharge nurse training with home visits and phone calls	- N. hospitalizations<br>- No. of deaths from COPD<br>- Quality of life (SGRQ).
**Billington et al. 2014**	United Kingdom	12 weeks	71 GI: 34 GC: 37	Randomized controlled trial	- Patients with COPD<br>- Previous spirometry results of FEV1/FVC ratio of 70% or less.<br>- Knowing how to read and speak English.	- Plan with telephone nursing support (intervention)<br>vs.<br>- Self-care plan only (usual care)	- Impact of COPD on quality of life:<br>- CAT<br>- Number of exacerbations<br>- Service satisfaction<br>- Hospital visits

**Bischoff et al. 2012**	Holland	24 months 2004-2006	165 GI:55 GC:55 Gx:55	RCT Randomized controlled trial	- Patients with COPD.<br>- Age > 35 years.<br>- FVC < 0.7.<br>Exclusion<br>-FEV1 <30%<br>- Therapy with a respiratory doctor<br>- With serious comorbid conditions<br>- Reduced life expectancy<br>- Inability to communicate in Dutch<br>- Objections to mode of management of disease used in study	- Usual care + self-management<br>vs.<br>- Usual care + routine monitoring by a primary care nurse	- Frequency and management of exacerbations<br>- Exacerbation assessment telephone system. (TEXAS).<br>- Dyspnea level: MRC.<br>- Quality of life assessment of people with COPD: CRQ.<br>- COPD self-sufficiency scale: CSES
**Cai et al. 2020 (10)**	China	12 months 2018	120 GI: 60 GC: 60	Retrospective study	- Patients with moderate COPD.<br>- FEV1/FVC <70%<br>- 50% < FEV1 < 80%	- Bi-directional quality feedback nursing model<br>vs.<br>- Conventional nursing intervention	- Quality of life:<br>- World Health Organization<br>- Quality of Life Scale Abbreviated<br>- Version WHOQOL-BREF.
**Cameron-Tucker et al. 2016**	Australia	8- 12 weeks	65 GI: 35 GC: 30	Randomized clinical trial with parallel groups	- Patients with COPD.<br>- Age > 18 years.<br>- Present an exacerbation at least 2 months before data collection.<br>- Excludes cognitive impairment	- Tele-rehabilitation<br>vs.<br>- Usual care	- Physical capacity: 6MWD.<br>- Impact of COPD on quality of life:<br>- CAT<br>- Health Behavior: SNAPPS

**Chau et al. 2012**	China	2 months	40<br>GI:22<br>GC:18	Randomized unblinded parallel group study	- Age > 60 years.<br>- Patients with moderate or severe COPD.<br>- Patients admitted to hospital at least once due to exacerbation during previous year.<br>- Excluding those with cognitive impairment, illiterate or unable to use tele-assistance technological instrument	- Use of telecare system with community nurse and.<br>- Usual care	- Satisfaction questionnaire.<br>- Quality of life assessment of people with COPD: CRQ.<br>- Lung function: FEV1 and FVC.<br>- Access to emergency service<br>- Or hospital admissions
**Cumming et al. 2010**	Australia	12 months	36<br>GI: 17<br>GC: 19	RCT Randomized controlled trial	- Patient with COPD.<br>- Age > 45 years.<br>- Patients with at least one exacerbation during previous year.<br>- Mini-mental scale > 21<br>- No palliative patient or with other respiratory diseases<br>- Owning a telephone<br>- Able to give informed consent	- Use of electronic monitoring techniques and tutoring by community nurses vs.<br>- Usual care	- Use of monitoring diary.<br>- Hospital admissions.<br>- Stanford Chronic Disease Management Scale

**De San Miguel et al. 2013**	Australia	6 months	71<br>GI:36<br>GC:35	RCT Randomized controlled trial	- Patients with COPD.<br>- Patients with O2 at home.<br>- Speaking English.<br>Exclusion:<br>- Patients with dementia<br>- Palliative care<br>- No telephone<br>- Cognitive impairment	- Remote monitoring of vital parameters with a telemedicine team assisted by a nurse.<br>vs<br>- Nursing assistance data collection of vital parameters only	- N. visits to general practitioner<br>- N. specialist medical visit<br>- N. access to Emergency Department<br>- N. hospital admissions<br>- N. days admitted
**Deng et al. 2013**	China	6 months 2010-2011	64<br>GI:32<br>GC:32	RCT Randomized controlled trial	- Patients with COPD.<br>- FEV1: 60-25% post bronchodilator<br>- Speaking Mandarin<br>Exclusion.<br>- Severe disability caused by other diseases	- Non-pharmacological therapy to better manage dyspnea by a nurse: psychological, cognitive-behavioral, physical-functional<br>vs<br>- Usual therapy	- Dyspnea level:<br>- MRC.<br>- MFI-20
**Doğan et al. 2017**	Turkey	5 months 2015	63<br>GI: 32<br>GC: 31	Quasi-experimental study	- Patients with COPD.<br>- Patients > 18 years old.<br>- Patients with prescription of oxygen concentrator with ATS and ERS criteria.	- Health education given by nurses to COPD patients on daily use of oxygen concentrator<br>vs.<br>- Absence of this training	- Spirometry.<br>- SaO2<br>- pH<br>- PaO2<br>- FEV1<br>- N. exacerbations in 3 months<br>- N. hospitalizations in 3 months.

							- Level of satisfaction in use of O2 concentrator
**Early et al. 2017**	United Kingdom	9 months 2012-2013	14 G1:8 G2:6	Case control study	- Patients with COPD<br>- FEV1 < 50%<br>- FVC < 0.7<br>- GOLD III, IV<br>- 2 COPD exacerbations related to hospital admissions in last year.<br>- Age >18 years<br>- Without pulmonary rehabilitation in last year.	- Internet-based standard program, TPP and support from nurse advisers (Group 1 focused on getting fit and staying out of hospital)<br>vs.<br>- Internet-based standard program, TPP and nurse-advisor support (focused on daily activities and slowing COPD progression).	- Assessment of quality of life for People with COPD:<br>- CRQ.<br>- Hospital Anxiety and Depression Scale: HADS.<br>- Need for patient information:<br>- Lung Information Needs Questionnaire LINQ

**Efil et al. 2020**	Turkey	12 months 2016-2017	59 GI:29 GC:30	Case control study	- Patients with COPD.<br>- Use of inhalers for at least 6 months.<br>- GOLD II, III Exclusive<br>- Patients with deafness, blindness or alcohol or drug dependence	- Inhaler training (oral explanation and a demonstration)<br>vs.<br>- Lack of training on inhalers	- Medication adherence:<br>- Morisky Medication Adherence<br>- Scale (MMAS-8).<br>- Quality of life (SGRQ).<br>- Impact of COPD on quality of life:<br>- CAT<br>- FEV1
**Heslop-Marshall et al. 2018**	United Kingdom	12 months	236 GI:115 GC:121	Randomized controlled trial	- Patients with COPD FEV1/FVC < 70%<br>- Patients with HADS – anxiety subscale scores of 8. ≥<br>- Agree to attend a minimum of two and a maximum of six CBT sessions. (Therapy)	- Cognitive behavioral therapy<br>vs.<br>- Regular intervention	- Quality of life related to health: EQ5D<br>- Anxiety and depression scale: HADS
**Iniesta Sánchez et al. 2016**	Spain	24 weeks 2007-2008	143 GI;56 GC:87	Quasi-experimental study pseudo random	- Patients with COPD	- Care plan with taxonomies<br>vs<br>- Regular hospital care.	- Evaluation and evolution of NOC<br>- Knowledge of therapeutic regimen
**Ingadottir et al. 2010**	Iceland	18 months 2006-2008	42	Pre-post intervention study.	- GOLD II, III, IV<br>- Hospitalized in last few months.<br>- Important change in health before and after hospitalization.	- Nursing intervention based on helping and improving life of patients with COPD and their families<br>vs.	- SGRQ quality of life.<br>- HADS Hospital Anxiety and Depression Scale.<br>- Body mass index BMI<br>- N. Hospital admissions<br>- Ability to use inhaler correctly

					- Be autonomous at home	- Regular intervention	
**Jolly et al. 2018**	United Kingdom	12 months	516<br>GI:239<br>CI:277	Randomized controlled trial	- COPD patients<br>- MRC 1/2 scale in primary care	- Telephone intervention of health training given by nurses:<br>- Smoking cessation.<br>- Increase physical activity<br>- Pharmacological management<br>- Action planning<br>vs.<br>- Intervention usually alone	- Quality of life (SGRQ).<br>- EQ- 5D-5L<br>- HADS anxiety<br>- HADS depression<br>- MRC dyspnea<br>- Hospital admissions<br>- Access to Emergency service<br>- Access to primary care service<br>- Smoking cessation<br>- IQR therapeutic adherence
**Jurado Gámez et al. 2012**	Spain	1 year<br>2010-2011	71<br>GI:36<br>GC:35	Randomized controlled trial	- Patients with COPD<br>- Age < 75 years<br>- Belonging to study health area<br>Excluded:<br>- Severe comorbidity<br>- No phone line	- Nursing home visit 48-72 hours after hospital discharge<br>vs.<br>- Usual care	- Arterial blood gases<br>- FEV1<br>- Borg scale<br>- Charlson index<br>- N. hospital admissions

**Karasu et al. 2020**	Turkey	8 months 2017	160 GI: 80 GC:80	Randomized controlled trial	- Diagnosed COPD for at least 6 months or more.<br>- No communication problems.	-Home care following Health Promotion Model + interventions according to demands<br>vs.<br>-No additional nursing care	- ASA scale (self-care)<br>- Life Attitude Profile (LAP)
**Khoshkesht et al. 2015**	Iran	3 months 2010-2011	66 GI:34 GI:32	Randomized controlled trial	- Patients > 65 years.<br>- Patients with moderate or severe COPD.<br>- Not participating in any respiratory exercise group in previous year.<br>- Excludes cognitive, cardiological and musculoskeletal impairment.<br>- Able to read and write Persian.	- Pulmonary rehabilitation applying Bandura technique self-efficacy theory<br>vs<br>- Routine nurse visits with weekly phone calls.	- CSES self-efficacy scale
**Lamers et al. 2010**	Holland	20 months 2003-2005	119 GI:58 GC:61	Randomized controlled trial	- Patients > 60 years.<br>- Patients with COPD or emphysema or chronic bronchitis<br>- Excludes major depression	- Nursing management of the (MPI) minimal psychological intervention<br>vs<br>- Standard nursing treatment	- BDI (Beck Depression Inventory).<br>- SCL (Symptom Checklist-90)<br>- SGRQ (Saint George Quality of Life)

**Lavesen et al. 2016**	Denmark	1 year 6 months 2010-2012	178 GI:101 GC:77	Randomized controlled trial	COPD patients with acute pneumonia Exacerbation.<br>Excluded:<br>- Cognitive impairment, dementia.<br>- Deafness.<br>- Not speaking Danish.	- Phone follow-up by a nurse to empower patient<br>vs<br>- Usual treatment	- Mortality rate.<br>- Patient assessment of COPD Management (%)
**Lee et al. 2015**	South Korea	6 months 2010-2011	151 GI:78 GC:73	Randomized controlled trial	- Patient with COPD<br>- Age 40-80 years<br>- Life expectancy > 6 months	- Nurse-led problem-solving therapy<br>vs.<br>- Usual care	- EPOC Self-Efficacy Scale (CSES).<br>- Jalowiec Coping Scale (JCS)<br>- Depressive symptoms (CES-D Radloff)
**Li et al. 2014**	China	6 months 2008-2009	112 GI: 56 GC:56	Case control study	- Patients diagnosed with COPD.<br>- FEV1/FVC < 70%<br>- FEV1% < 80%<br>- Capacity for self-care during established period	- Usual care + 3 months follow-up<br>vs.<br>- Usual care	- Quality of life: Saint George respiratory questionnaire (SGRQ)<br>- Anxiety, depression: Goldberg's General Health Questionnaire (GHQ-28)<br>- Body Mass Index (BMI)
**Li et al. 2015**	China	19 months 2012-2013	61 GI:31 GC:30	Randomized controlled trial	- Patients with COPD<br>- FEV1 / FVC < 70%<br>- No change in preceding 4 weeks of pharmacological or symptomatic therapy<br>- Ability to speak Mandarin or	- Education in management of disease by an expert hospital nurse.<br>vs<br>- Usual care	- Quality of life:<br>- Seattle Obstructive Lung Disease Questionnaire<br>- COPD Self Efficacy Scale<br>- Frequency of exacerbations<br>- Frequency of emergency hospitalizations

					Cantonese<br>- Ability to use mobile phone.		
**Li et al. 2020**	China	4 months 2017	70<br>GI:35<br>GC:35	Randomized controlled trial	- Patients with COPD.<br>- GOLD I, II, III or IV<br>- Exposure to risk factors of different types<br>- FEV1/ FVC < 70%<br>- Ability to communicate in Chinese.<br>- Capable of completing questionnaires and participating in interviews.<br>- Access to use of a mobile phone	- Nursing care based on Information theory,<br>- Knowledge, Attitude and Practice IKAP<br>vs.<br>- Standard nursing care.	- Quality of life:<br>- Saint George Respiratory Questionnaire (SGRQ)
**Mohammadi et al. 2015**	Iran	4 months	40<br>GI:20<br>GC:20	Control case study	- Patients with COPD.<br>- GOLD II, III<br>- Not having other chronic diseases.<br>- No use of oxygen when performing daily activities<br>- No use of tranquilizers or antidepressants.	- Home rehabilitation of three x one-hour sessions of individual face-to-face training<br>vs.<br>- Absence of this service	-Fatigue level: fatigue severity scale (FSS).<br>- Capacity for activities of daily living:<br>- Barthel index.<br>- QOL quality of life

**Nguyen et al. 2009**	USA	6 months	17 Trained Assig,: 9 Monit.:8	Randomized exploratory study	- Patients with COPD.<br>- Severe condition according to GOLD criteria.<br>- FEV1/FVC<70%<br>- FEV1% < 80%<br>- Patients receiving supplemental oxygen were acceptable if their O2 saturation was maintained at 88% on 6 L/min nasal oxygen during six-minute walk test (6MW);<br>- Age > 40 years.	- Exercise via mobile (Long-term exercise support mobilization) alone vs.<br>- Exercising using a mobile phone (Support mobilization for long-term exercise) with help of a nurse	- For exercise regulation: Self-Regulation Questionnaire-Exercise [SRQ-E]) and Patient Activation Measure (PAM).<br>- Self-efficacy to overcome of barriers to exercise:<br>- Barrier effectiveness scale.<br>- General perception of support for exercise: Likert scale<br>- Functional capacity: 6-minute walk test (6MWT).<br>- Free-living ambulatory physical activity using a Stepwatch activity (buckled to ankle).<br>- Health-related quality of life (HRQoL)<br>- Quality of life: Saint George respiratory questionnaire (SGRQ)

**Padilla-Zárate et al. 2013**	Mexico	1 year	60<br>GI: 35<br>GC: 25	Randomized controlled trial	-Patients with COPD,<br>-GOLD I, II, III or IV	- Medical care and IEBCP (nursing-intervention based on personalized counseling)<br>vs.<br>- Medical care	- Self-care.<br>- Quality of life: St. George respiratory questionnaire and quality of life questionnaire in respiratory disease (CQR).<br>- Oximetry: oximeter.<br>- Relationship between expired volume maximum in first second of forced expiration and forced vital capacity: spirometer.<br>- Dyspnea: Medical Research Council dyspnea scale.<br>- Adherence to intervention.
**Saleh et al. 2014**	Norway	1 year<br>2010-2011	56	Retrospective cohort study	- Patients with COPD<br>- Age> 40 years<br>- Not having other serious illnesses with a life expectancy of less than 12 months.<br>- Ability to communicate	- Impact of use of video consultations on hospital readmission for exacerbations and patient satisfaction	- Number of re-admissions due to exacerbations<br>- Frequency and duration of these hospitalizations<br>- Patient satisfaction

**Scheerens et al. 2020**	Belgium	6 months	25<br>GI: 13<br>GC: 12	Randomized controlled trial	- Patients with COPD<br>- Oxygen dependent.<br>- Three or more hospitalizations for COPD in last three years<br>- CAT scale<br>- Scale 4 dyspnea according to MRC.<br>- Intubation in last year<br>- Non-invasive ventilation in last year<br>- BMI = 18<br>- NYHA functional classification 3	- Integrated palliative home care<br>(COPD pre-inclusion support training, monthly visits, brochures on coping mechanisms, a protocol on symptom management and support, action plan and care plan, integration of project and care through complaint and communication mechanisms)<br>vs.<br>- Usual care	-Health-related quality of life: HRQOL<br>-Mood and anxiety: Hospital Anxiety and Depression Scale (HADS)<br>- Impact of COPD on quality of life: CAT<br>- Number of exacerbations, primary care interventions, hospitalizations and living will decisions, measured with patient-reported Health records.<br>- Evaluation of care received: Dutch Patient Assessment of Chronic Illness Care (PACIC)<br>- Place of death: medical care involved.
**Shany et al. 2017**<br>**(37)**	Australia	1 year<br>2009-2010	29<br>GI:11<br>GC:18	Randomized controlled trial	- Patients with COPD<br>- Sydney resident patients treated by Blacktown Hospital.<br>- At least one hospital admission for a COPD exacerbation in previous year	- Monitoring of oximetry, temperature, pulse, electrocardiogram, blood pressure, spirometry and weight with telephone support and home visits<br>vs.	- Quality of life: Saint George Respiratory questionnaire (SGRQ)<br>- Mood and anxiety: Hospital Anxiety and Depression Scale (HADS)<br>- Number of hospital admissions and length of stay.

						- Only telephone support and home visits	
**Sorknæs et al. 2011**	Denmark	1 year 2008-2009	100 GI:50 GC:50	Randomized controlled trial	- Patients with COPD<br>- Exacerbations with dyspnea, cough and/or sputum.<br>- Need to increased medication.	- Use of tele-consultations between COPD patients and respiratory nurses	- Health-related quality of life: EQ- 5D<br>- Body mass index: BMI<br>- Dyspnea: MRC<br>- FEV1/FVC, FVC and FEV1: spirometry
**Sorknaes et al. 2013**	Denmark	1 year	242 GI: 121 GC:121	Randomized controlled trial	- Patients with COPD by spirometry FEV1/FVC <70%<br>- Admitted with exacerbations (defined by increased need for medication and increased dyspnea, increased volume of expectoration or increased cough)<br>- Age<40 years	- Daily teleconsultations in real time for one week between hospital nurses specialized in respiratory diseases and patients with severe COPD discharged after an acute exacerbation vs.<br>- Usual care	- Re-admissions to hospital<br>- Length of hospital stay<br>- Body mass index<br>- Dyspnea: MRC<br>- Spirometry<br>- Spanish health questionnaire: SF-36<br>- Need for home care<br>- Mortality

**Utens et al. 2012**	Holland	3 months	125<br>GI:63<br>GC:52	Randomized controlled trial	- Patients with COPD<br>- Minimum GOLD I<br>- 10 packs smoked per year<br>- Hospitalized for exacerbations.<br>- Age < 40 years	- Discharge assisted by community nurses (after hospitalization for exacerbations)<br>vs.<br>- Usual hospital care	- Change in health status: Clinical COPD Questionnaire (CCQ).<br>- Health-related quality of life: HRQL<br>- Mortality<br>- Readmissions<br>- Treatment failures
**Utens et al. 2014**	Holland	3 months	125<br>GI:63<br>GC:52	Randomized controlled trial	- Patients with COPD<br>- Age < 40 years<br>- Hospitalized for exacerbations	- Hospital care at home<br>vs.<br>- Usual hospital care	- Change in health status: Clinical COPD Questionnaire (CCQ).<br>- Caregiver stress: Caregiver Stress Index (CSI).<br>- Caregiver satisfaction: a series or questions<br>- Preference of users
**Wang et al. 2014**	China	3 months	88<br>GI:42<br>GC:46	RCT<br>Randomized controlled trial	- Patients with COPD<br>- Age < 45 years<br>- FEV1 /FVC: 70%<br>- FEV1 between 30-80%<br>- Not participating in any other research or habilitation program.<br>-Being able to communicate in Chinese	- Model of beliefs in health after hospitalization<br>vs.<br>- Routine nursing care	- Dyspnea: MRC<br>- Ability to perform basic activities of daily life: Barthel.<br>- Pulmonary functions: FEV1 and FVC<br>- Exercise Tolerance: 6MWT<br>- Self-efficacy of patients with COPD: COPD Self-Efficacy Scale (CSES)

**Wang et al. 2018**	China	1 year<br>2016 - 2017	120<br>GI: 60<br>GC: 60	Randomized controlled trial	- Patients diagnosed with COPD.	- Humanistic nursing care<br>vs.<br>- Regular nursing care	- Degree of anxiety and depression:<br>-Zung Self-Assessment Scale for Depression (SDS) and Zung Self-Assessment Anxiety Scale (EAA)<br>- Pulmonary function: FVC, MMF and FEV1.<br>- Nursing degree of satisfaction: self-designed questionnaire
**Wang et al. 2020**	China	1 year	154<br>GI:77<br>GC:77	Randomized controlled trial	- Patients with COPD<br>- GOLD and II, III or IV<br>- Patients hospitalized for acute exacerbation of COPD	- Nurse-led self-management program<br>vs.<br>- Usual care	- Exercise Tolerance: 6MWT<br>- Number of hospitalizations, length of stay and visits to emergency ward.<br>- Quality of life: Saint George respiratory questionnaire (SGRQ)<br>- Patient satisfaction: COPD Transitional-Care Patient Satisfaction Questionnaire (CTCPSQ)
**Wood-Baker et al. 2012**	Australia	12 months	69<br>GI: 36<br>GC:33	Randomized controlled trial	- Patients with COPD<br>- Patients with acute exacerbation<br>- Living in designated areas<br>- Age > 45 years.<br>- History of smoking. 10 pack-years.<br>- FEV1 /FVC: 0.7	- Program to increase self-care behaviors taught by community health nurses<br>vs.<br>- Usual care.	- Health-related quality of life: SF-36<br>- Patient self-efficacy: Stanford Self-Efficacy scale<br>- Number of medications at discharge and comorbidity: geriatric index of comorbidity.<br>-Weight.

					- At least one COPD exacerbation in previous 12 months<br>- Meeting requirements of study (Mini-Mental score greater than 21)<br>- Access to computer and telephone.		
**Yu et al. 2014**	China	1 year<br>2011-2012	84<br>GI: 42<br>GC: 42	Quasi-experimental study	- Patients with COPD<br>- FEV1 <70%<br>- Age > 40 years<br>- Able to read and write Chinese<br>- Lucid and oriented<br>- Able to use phone	- Self-management of education<br>vs.<br>- Usual care without additional education	- Quality of life: Saint George Respiratory questionnaire (SGRQ)
**Zakrisson et al. 2016**	Sweden	3 years	64<br>GI:40<br>GC:24	Quasi-experimental study	- Patients with COPD<br>- GOLD II, III<br>- Age 60-75 years	- Training at home by a nurse to quit smoking<br>vs<br>- Usual care without additional education	- Exercise Tolerance: 6MWT<br>- Quality of life: Saint George respiratory questionnaire (SGRQ)<br>- No COPD exacerbations
**Zakrisson et al. 2020**	Sweden	1 year<br>2015-2016	202<br>GI:94<br>GC:108	Randomized controlled trial	- Patients with COPD	- Usual care + informative self-management support<br>vs.<br>- Usual care only	- Quality of care: Patient's Perspective questionnaire (QPP)<br>- Impact of COPD on patient's life: COPD Assessment Test (CAT)

HAD Anxiety

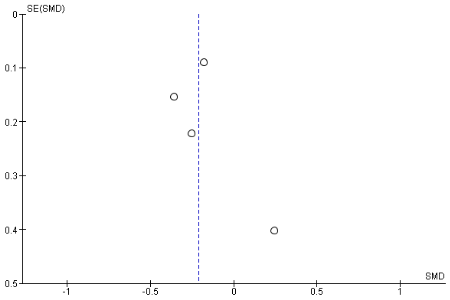

Barthel Score

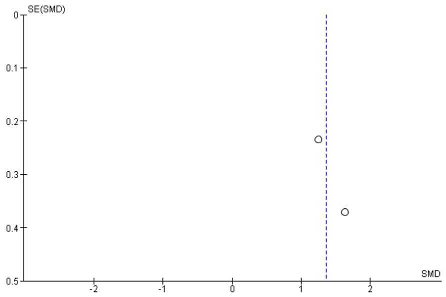

6 MWT Test

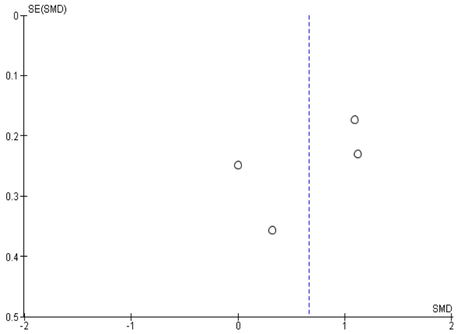

Figure S1. Assessment of publication bias. Funnel Plots.

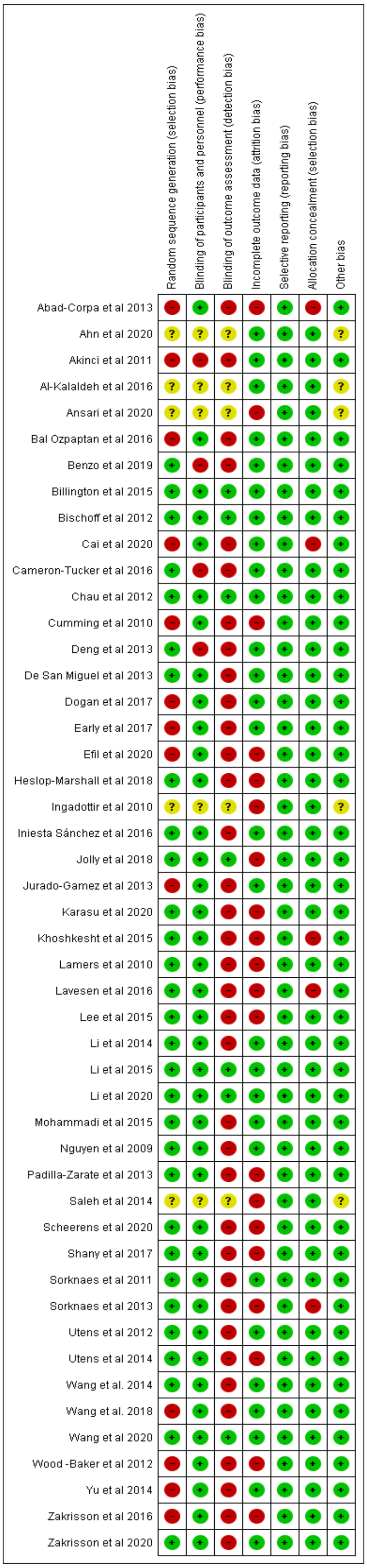

Figure S2. Risk of bias of all selected studies.
